# Supplementary material for: Substitutional Coinage Metals as Promising Defects for Adsorption and Detection of Gases on MoS2 Monolayers: A Computational Approach
Source: Int J Mol Sci. 2023 Jun 17;24(12):10284. doi: 10.3390/ijms241210284 (PMC10299679; doi:10.3390/ijms241210284)
Supplement: Supplementary file 1 [file ijms-24-10284-s001.zip › ijms-2406454-supplementary.pdf]

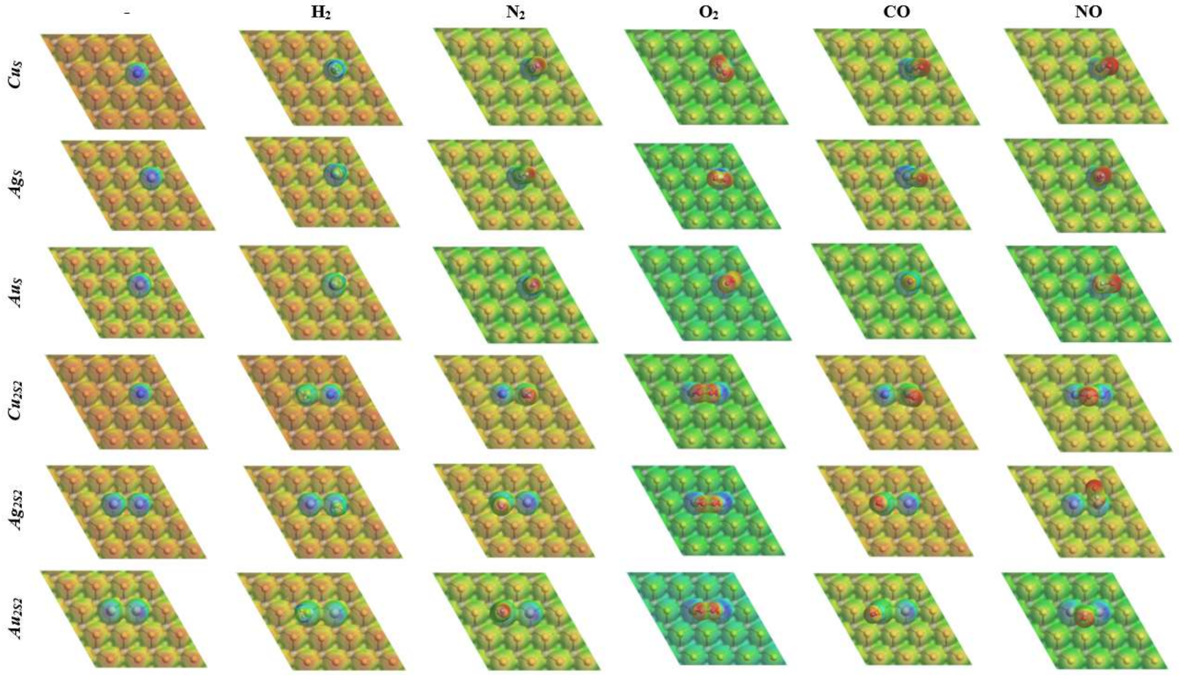

Figure S1. Electrostatic potential maps, mapped on isosurfaces with 0.004 a.u. of electron density, for defective MoS_2 monolayers interacting with atmospheric components (H_2, N_2 and O_2) as well as pollutants (CO and NO) in their ground states.

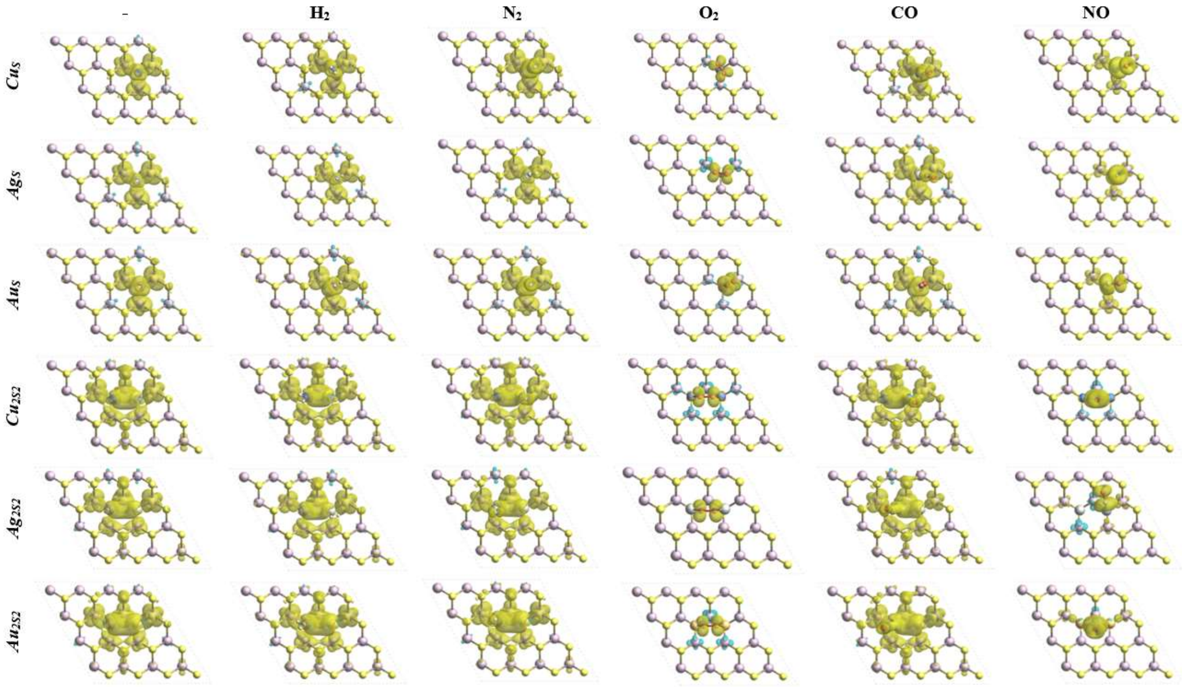

Figure S2. Spin density, mapped on isosurfaces with 0.004 a.u. of electron density, for defective MoS_2 monolayers interacting with atmospheric components (H_2, N_2 and O_2) as well as pollutants (CO and NO) in their ground states.

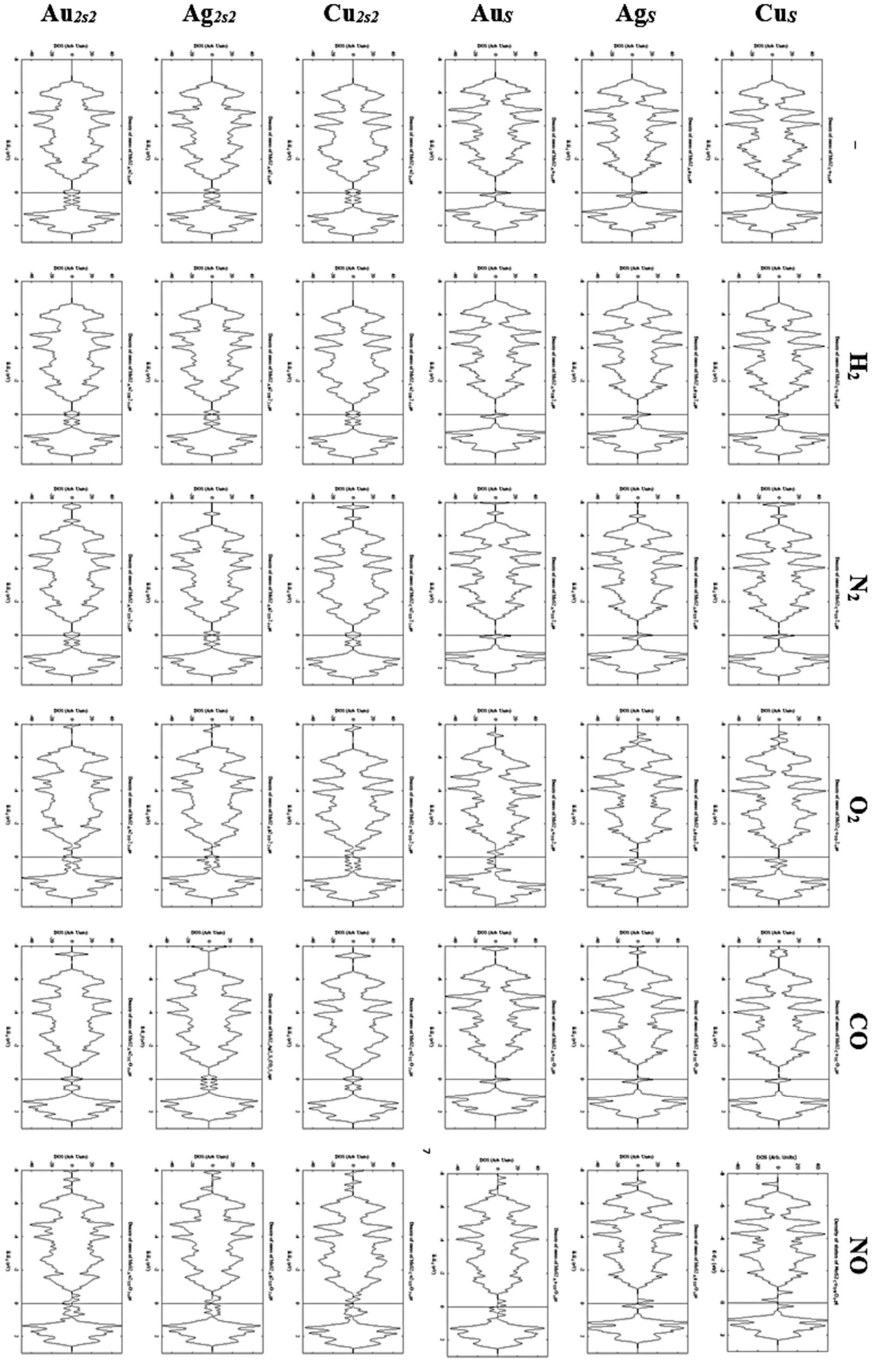

Figure S3. Density of states for the defective systems are shown. Fermi energy set at 0 eV.

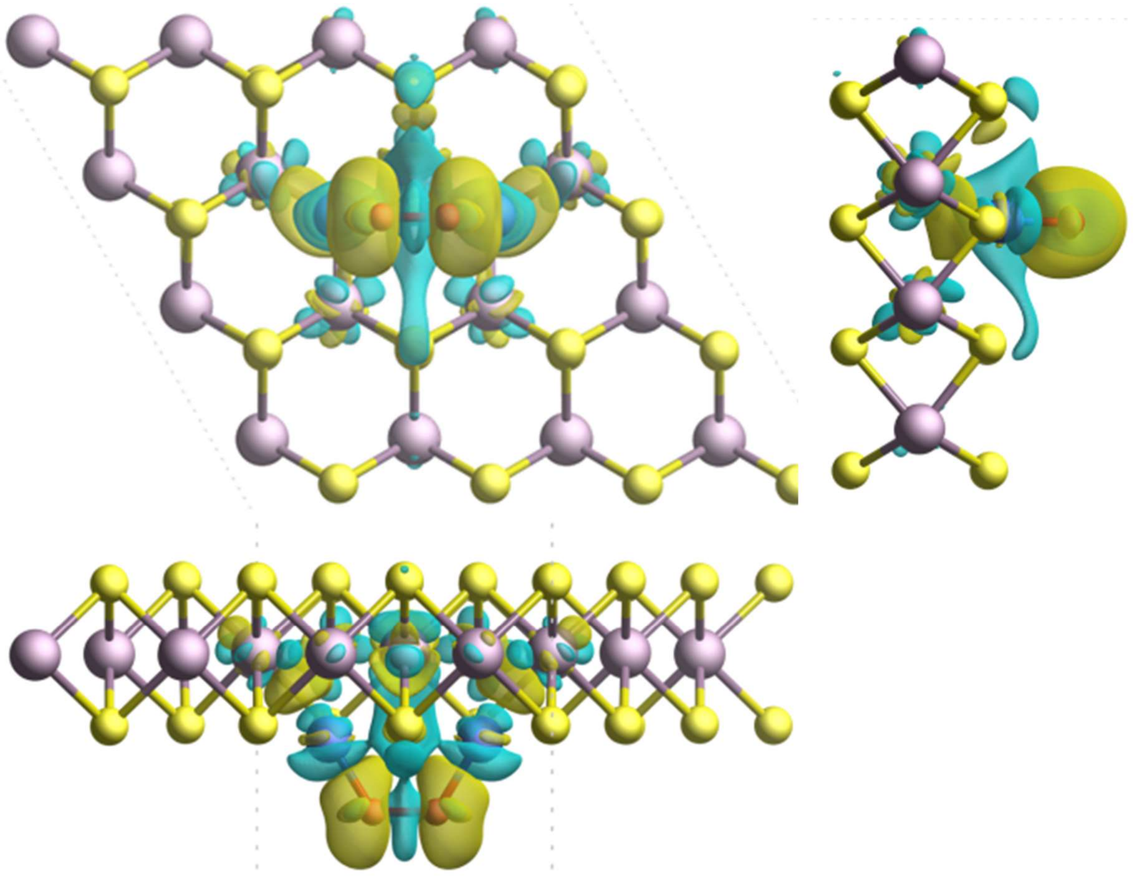

Figure S4. Charge density difference, mapped on isosurfaces with 0.004 a.u. of electron density, for MoS_2-Cu_{2S2}-O_2 system. Three perspectives are shown.
